# Stakeholder Perceptions of Welfare Issues and Indicators for Extensively Managed Sheep in Australia

**DOI:** 10.3390/ani7040028

**Published:** 2017-03-23

**Authors:** Amanda K. Doughty, Grahame J. Coleman, Geoff N. Hinch, Rebecca E. Doyle

**Affiliations:** 1 Animal Science, University of New England, Armidale NSW 2350, Australia; ghinch@une.edu.au; 2 CRC for Sheep Industry Innovation, Armidale NSW 2350, Australia; 3 Animal Welfare Science Centre, The University of Melbourne, Parkville 3010, Australia; grahame.coleman@unimelb.edu.au (G.J.C.); rebecca.doyle@unimelb.edu.au (R.E.D.)

**Keywords:** animal welfare, attitudes, gender, general public, producers, survey, welfare indicators, welfare issues

## Abstract

**Simple Summary:**

This survey was designed as the first step in the development of a welfare assessment framework with the aim of identifying potential causes of welfare compromise and useful indicators for sheep in extensive Australian production systems. We asked the general public, sheep producers, service providers and sheep industry related scientists to provide their thoughts on the importance of a range of sheep welfare issues and possible key indicators. All respondents thought sheep welfare was adequate but that improvement was desired. Issues perceived to cause the most risk to sheep related to factors influenced by the environment (i.e., nutrition and food supply), heat stress and lameness while key indicators useful to assess welfare were nutrition and food availability, mortality/management issues, pain and fear related indicators, and numbers of illness/injuries. Women and the general public perceived all issues and indicators to be more important than other groups of respondents. These results highlight the need to consult a wide range of stakeholders in order to develop a broadly acceptable assessment system.

**Abstract:**

An online survey was designed to form the basis of a framework for the welfare assessment of extensively managed sheep in Australia. The survey focused on welfare compromise and useful welfare indicators. A total of 952 people completed the survey in its entirety, representing four stakeholder groups: Public (53.6%), Producer (27.4%), Scientist (9.9%), and Service provider (9.1%). Animal welfare was considered to be important by all participating groups in this survey (average score of 3.78/4). Respondents felt the welfare of grazing sheep was generally adequate but improvement was desired (2.98/5), with female members of the public rating sheep welfare significantly worse than other respondents (*p* < 0.05). Environmental issues were considered to pose the greatest risk to welfare (3.87/5), followed by heat stress (3.79), lameness (3.57) and husbandry practices (3.37). Key indicators recognised by all respondents were those associated with pain and fear (3.98/5), nutrition (4.23), mortality/management (4.27), food on offer (4.41) and number of illness/injures in a flock (4.33). There were gender and stakeholder differences in the perceived importance of both welfare issues and indicators with women and the public consistently rating issues (all *p* < 0.01) and indicators (all *p* < 0.05) to be of greater significance than other respondents. These results highlight the importance of including all stakeholders and an even balance of genders when developing a welfare framework that can address both practical and societal concerns.

## 1. Introduction

Extensive sheep production systems differ considerably from more intensive systems and sheep tend to be maintained on pasture outdoors year round and provided supplementary feed as required. The welfare of sheep in these systems is generally considered to be high [1]. This is largely due to sheep being perceived to be able to express natural behaviours in the farm setting. Three other drivers are also attributed to this perception: (1) the general public assumes that extensive conditions equal positive welfare; (2) issues faced in extensive production are perceived by producers as being ‘natural’ and therefore are either not easily addressed, and/or important to address; and (3) there is a belief that sheep have evolved in these extensive conditions, and so are well adapted to the environment in which we manage them [1].

However, the extensive management that creates a positive perception of sheep welfare may also create welfare risks. Extensively managed sheep are at risk of climatic extremes, predation and variable nutrition. Their contact with humans can be limited, and so any contact that does occur is likely to be associated with fear and distress, and this also reduces the ability to identify and treat health problems [1,2]. One comprehensive study that gauged the opinions of sheep experts [3] identified welfare issues for sheep in extensive UK settings that incorporate all of the Five Freedoms: 14 issues were associated with hunger and thirst; 14 with discomfort; 11 with pain/injury/disease; 8 with ability to express normal behaviour; and 10 with fear and distress. This suggests that potential compromises to welfare do exist and are worth monitoring.

The scientific assessment of animal welfare provides objective evidence about what an animal is experiencing, has experienced or is likely to experience. While such assessment is complex, decisions about what are acceptable levels of animal welfare require ethical judgements that underlie determining what is the “right” or “wrong” way to treat an animal [4]. A variety of factors influence a person’s perceptions of an animal’s welfare, much of which are driven by their attitudes and beliefs [5] and consequently perceptions about sheep welfare are likely to differ significantly based on who is asked. Expert opinion has been commonly used to identify welfare issues for the sheep industry [3,6], as well as providing information on key indicators [7], and has given a meaningful focus for research and extension programs. Consulting more broadly also has its benefits. Producer perspectives of welfare are arguably the most important [8,9,10] as they can identify a breadth of welfare issues seen on their farms, and have the day-to-day experience and control over the management of their animals. The perceptions that the general public have about welfare are also important as they consume the products and give the industry its “social licence” [11], or public trust [12]. Public attitudes and values are also the drivers for animal welfare improvements, particularly the level of cognition and consciousness attributed to a species [13], and while progress into understanding these differences has been made, there is still much more to be understood, including how citizens consider and assess animal welfare [14].

Surveys about extensively managed sheep have been conducted on individual stakeholders [3,15,16], as have surveys about intensive sheep management across multiple stakeholder groups [17]. To our knowledge, the only survey investigating welfare issues across stakeholder groups for extensively managed sheep was conducted by Phillips et al. [18]. This survey assessed perceptions on welfare issues and compared welfare scenarios in sheep, goats and beef cattle, targeting specific stakeholder groups including animal rights advocates [18], but did not survey the general public. There has also been little research on suitable indicators of sheep welfare in extensive systems. Work done by Phythian et al. [3] on welfare indicators for sheep in a UK setting reported that there is a wide range of possible indicators that could be used in on-farm assessments and some of these have been utilised in a recently released welfare assessment protocol [19]. Suitable indicators were identified through expert opinion groups; however it remains unclear how acceptable they are to both the producers, who will be utilising them, and the general public. While it is likely that the general public has little interest in developing a detailed knowledge of animal management and husbandry practices [17,20], areas of agreement between the various stakeholder groups toward suitable key indicators should be considered along with reliability, validity and feasibility when developing a welfare assessment framework.

With this in mind, the current study was designed to form the basis of a comprehensive framework for the welfare assessment of extensively managed sheep in Australia, thus adding to the work outlined above. We did so by surveying interested stakeholders to identify what they considered to be the important risks to sheep welfare and key indicators of welfare state. Based on previously published evidence, it was hypothesised differences would exist between stakeholder groups [16,17,18]. Having a better understanding of perceptions of the welfare of extensively managed sheep can help to focus research needs and targeted engagement for stakeholder groups. This can also be useful information for future animal welfare standards and legislations.

## 2. Materials and Methods

This study received ethics approval from the University of Melbourne’s School of Land and Environment Human Ethics Advisory Group on 14-11-2014 (Ethics ID: 1443082).

### 2.1. Survey Description

A large survey was conducted to obtain information about people’s perceptions of extensively farmed sheep in Australia. The final format of the survey was determined through extensive consultation with an advisory group of 10 that comprised experienced animal welfare scientists (*n* = 3), industry advisors (2), sheep extension officers (2) and sheep producers (3). The survey was conducted online and was developed using the online survey platform Survey Monkey [21]. Once participants had read through the introduction, they selected which stakeholder category best described them: sheep farmer (producer), sheep industry service provider or advisor (service provider), sheep focused scientist/researcher (scientist), general public, or other. While all stakeholders answered a set of 11 common questions, some requiring multiple responses, participants also answered additional questions tailored specifically for that stakeholder group category. Questions covered perceived welfare issues, perceptions of welfare indicators and self-rated knowledge, along with basic demographic questions. The question formats varied with the question and involved: (1) respondents selecting a single choice from the range provided; (2) selecting the most appropriate response on a Likert scale; and (3) replying freely to an open-ended question (Table 1). Likert scales were 1–5 for all questions except for the question “What is your belief about animal welfare?”, which had a scale of 1–4.

### 2.2. Survey Distribution

The survey was accessible from the 8 December 2014 to the 8 January 2015 inclusively. The link to the survey was distributed through a variety of sources, aimed at encouraging interested parties to participate. There was no reward/incentive for participating.

The survey was distributed throughout Australia in a variety of ways ranging from subscribers of the Cooperative Research Centre for Sheep Industry Innovation (Sheep CRC) news bulletin (which has 6000 subscribers, no prerequisites for joining, and is made up of producers, industry service providers and others with an interest in sheep production) to the Facebook pages of RSPCA Australia (both state and national) and to 15 research institutions/universities/state government agricultural organisations.

### 2.3. Data Management and Statistical Analyses

All data were managed and analysed using the statistical program R (R Core Team, Vienna, Austria) [22]. Before analysis, responses were assessed for legitimacy. Four responses scored the maximum or minimum for all questions, and none of these gave comments/further information about their perceptions, so they were deemed uninformative and were removed from the dataset. Only the results from people who had completed the survey in its entirety were used for the analysis, and 88 respondents that identified themselves as ‘other’ were able to be reallocated to one of the four stakeholder groups for statistical analysis based on the written description they gave of their role in the sheep industry. Where details were insufficient the record was deleted.

Principal Components Analyses (PCA) with Oblimin rotations were used to summarise the 20 welfare issues and the 17 welfare indicators into meaningful components. PCA is widely used to assist in classifying terms that have been measured using Likert scales [23]. The criteria for classification of components were that the variable had to have a loading on the relevant component of at least 0.34 and must not load on more than one component [24]. This resulted in two components for the welfare issues and two issues remained independent of the components; for the welfare indicators, three components were generated and two indicators remained independent (Table 2). Composite scores for the components were then calculated by adding the Likert scores for each of the variables in the component and taking the average.

Parametric analyses were used for the Likert Scale data, which is a common way to manage survey data, particularly in the case of composite scores that arise from PCA [23]. Analyses of variance (ANOVA) were used to analyse differences between stakeholders and gender, with these factors (stakeholder and gender) and their interaction included as independent variables [25]. An ANOVA was performed to investigate differences between the levels of self-rated knowledge the general public attributed to the different questions, and post-hoc comparisons were performed using least significant differences. Post-hoc comparisons were performed using Tukey’s tests in the program Agricolae. Pearson’s correlations were conducted to investigate relationships between demographics, welfare beliefs and self-rated industry knowledge, and Spearman’s rank correlations were used to investigate the relationship between education (an ordinal variable) and beliefs about animal welfare and sheep welfare. Correlation values were classified as strong if coefficients were ≥ 0.5 and moderate if between 0.3 and 0.49 [26]. 

## 3. Results

### 3.1. Demographics

A total of 1535 people responded to the survey during the month it was open, and 956 of these completed the survey in its entirety. Of these, 15 were under 18 years of age. While the survey was voluntary, the ethics application only covered participants 18 years or over, and so these 15 were removed from the results. The breakdown of the 941 valid participants according to stakeholders was: General public = 499 (53.0%), Producer = 260 (27.6%), Scientist = 95 (10.1%), and Service provider = 87 (9.2%). The genders of the respondents were skewed towards women (61.2% overall), and a difference within each stakeholder category existed; the percentages of women represented in each group were: 27% for producers, 45% scientists, 25% service providers and 88% general public. The majority (70%) of survey respondents were tertiary educated, (a higher proportion than the 44% of Australians in the general population [27]) with others being trained at a technical institute (19.0%) or high school (11%). Participants age ranged from 18 years old to 82 years old, with both an average age and median age of 47 (1967) and the mode was 58 (1956).

All of Australia’s states and territories were represented, with the largest portion coming from New South Wales (30.8%) and then Victoria (27.6%), South Australia (15.3%), Western Australia (10.8%), Queensland (9.9%), Tasmania (3.3%), the Australian Capital Territory (1.5%) and the Northern Territory (0.8%); with these proportions being roughly equitable with the overall distribution of the population. The general public, sheep industry service providers and sheep-specific scientists were asked to select their current residential location, with producers all being classified as rural and not included below. The majority of respondents were suburban (27.2%) and 14.2% were rural, followed by urban (11.4%), country town (9.5%), regional city (7.0%), peri-urban (3.5%) and remote (0.3%); which was again roughly equitable with the overall distribution of the population, except for rural respondents, which was higher and reflective of the agricultural focus of this survey.

### 3.2. Welfare Issues

The majority of respondents considered animal welfare to be of major importance and women scored significantly higher than men on this response (F_1_ = 21.66, *p* < 0.001; Table 3). All of the 20 issues presented to respondents were identified as posing some degree of risk as all were above the midpoint of 2.5/5. Flystrike (4.25/5), nutrition (4.07) and predation (3.96) were perceived to be the greatest welfare issues; shearing (3.28), yarding (3.01) and the use of sheep dogs (2.87) were the least. While these three issues were considered to be of less risk, they were still above the scale midpoint.

**Figure 1:**
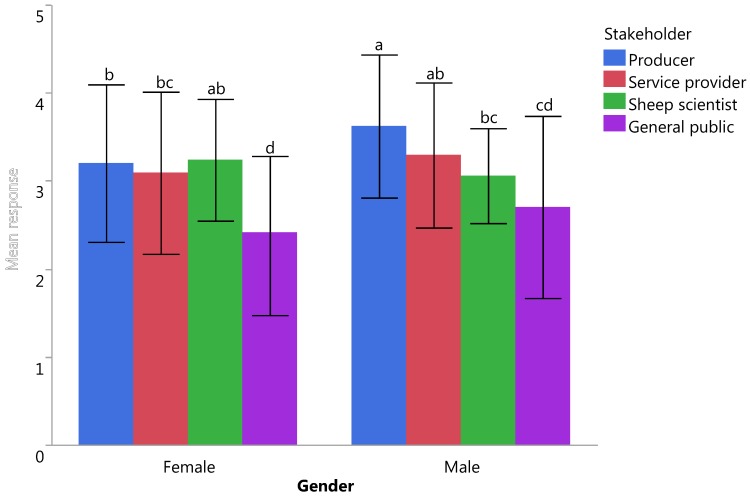
Belief about the welfare of grazing sheep in Australia according to different stakeholders and genders with a significant interaction present between gender and stakeholder (*p* < 0.05); survey responses could range from 1 = very poor to 5 = excellent; differences between letters (**^a–d^**) indicate statistically different means at *p* < 0.05; bars represent standard deviations.

The PCA identified four broad issues and, when analysing the composite scores derived from the PCA, environmental issues scored the highest and husbandry practices scored the lowest, and the scores were significantly different from each other (F_3_ = 37.52, *p* = 0.000). Gender differences were noted for all welfare issues (all *p* < 0.05), with women consistently considering the risk of welfare compromise to be higher than men (Table 3). Stakeholder also significantly influenced the results, with the general public considering welfare to be more important than the other three groups (F_3_ = 13.20, *p* < 0.001; Table 4). Respondents felt that the welfare of grazing sheep was generally adequate, but with improvement required, with a significant interaction between gender and stakeholder (F_3_ = 2.65, *p* = 0.048; Figure 1).

A minor, negative correlation was present between the two welfare belief-related questions (*r* (928) = −0.29, *p* < 0.001), indicating that as belief about the importance of animal welfare increases, perception of the welfare of grazing sheep decreases. There were no statistically significant relationships between education and animal welfare beliefs (*r* (955) = −0.056, *p* = 0.08), or the perception of grazing sheep welfare (*r* (929) = 0.048, *p* = 0.88), nor were there for age and animal welfare beliefs (*r* (952) = 0.015, *p* = 0.65), or age and the perception of grazing sheep welfare (*r* (926) = 0.026, *p* = 0.44).

Following questions where respondents were asked to consider specific welfare issues, they were also asked to list the three welfare issues they perceived to be most important. This was an open-ended question provided to all participants and they could respond by either using issues from previous questions and/or include other issues they considered to be important. For position 1 (the most important welfare issue) the frequent responses were “Live export” (*n* = 189, 19.9%), followed by “Mulesing/wrinkle score of the sheep/alternatives to mulesing” (*n* = 124, 13.0%) and then “Flystrike” (*n* = 85, 8.9%). For position 2 (the second most important welfare issue) the frequent responses were “Mulesing/wrinkle score of the sheep/alternatives to mulesing” (*n* = 90, 9.5%), “Environmental comfort” (heat stress, inadequate shelter, cold stress, space; *n* = 72, 7.6%) and “Pain, painful/stressful husbandry practices, stress and pain management” (*n* = 68, 7.1%). In position 3 (the third most important welfare issue) the most frequent replies were “Nutrition” (adequate access to food, stocking rates, improving nutritional values of pasture, overstocking feed supply; *n* = 76, 8.0%), “Predation and predator attacks” (*n* = 67, 7.0%), “Pain, painful/stressful husbandry practices, stress and pain management” (*n* = 66, 6.9%) and “Environmental comfort” (heat stress, inadequate shelter, cold stress, space; *n* = 65, 6.8%). These open-ended responses were then summarised into 7 categories: disease and illness, environmental, injury and painful husbandry practices, management, nutrition, off-farm and other to enable visual comparisons (Table 5). Numerical differences of open-ended welfare issues were evident between stakeholders and genders. Male producers consistently identified disease/illness as the most important issue for sheep welfare and the general public consistently identified off-farm or injury/painful husbandry practices as the most important welfare issues. Variability existed within the other groups; scientists identified disease/illness as the most important issue, followed by injury/painful husbandry practices and there was a gender split between these two categories for the third most important issue. Industry service providers swapped between disease/illness and injury/painful husbandry practices with differences between the genders. Female producers had the greatest range of issues, with the most important issue being disease/illness, the second most important issue listed was off-farm and the third most was injury/painful husbandry practices.

### 3.3. Welfare Indicators

A total of 17 possible welfare indicators were presented to the participants and they were able to rank each indicator with a score ranging from 1, unimportant when assessing the welfare of a sheep, through to 5, essential when assessing welfare.

**Figure 2:**
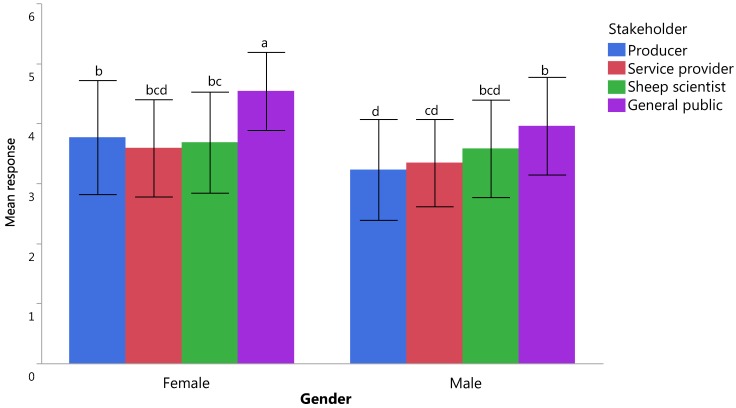
The importance of including welfare indicators relating to pain and fear in an on-farm assessment according to different stakeholders and genders with a significant interaction present between gender and stakeholder (*p* < 0.05); responses ranged from 1 = unimportant to know to 5 = essential to know; differences between letters (**^a–d^**) indicate statistically different means at *p* < 0.05; bars represent standard deviations.

**Figure 3:**
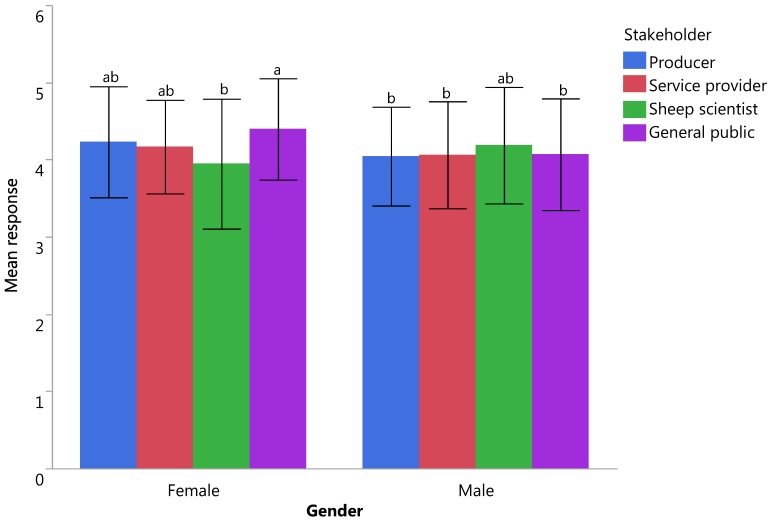
The importance of including welfare indicators relating to nutrition in an on-farm assessment according to different stakeholders and genders with a significant interaction present between gender and stakeholder (*p* < 0.05); responses ranged from 1 = unimportant to know to 5 = essential to know; differences between letters (**^a,b^**) indicate statistically different means at *p* < 0.05; bars represent standard deviations.

Three major components accounting for 15 variables and two independent indicators were identified following PCA. Of the composite scores derived from the PCA, feed on offer was found to have the highest mean score (4.40 ± 0.02) while the pain and fear related indicators had the lowest (3.98 ± 0.03) and the scores were significantly different (F_4_ = 36.89, *p* = 0.00). There were significant gender differences between the perceived importance of management issues, food on offer and numbers of illness/injuries (all *p* ≤ 0.05), with females consistently rating these welfare indicators as more important than males (Table 3). Respondents felt that indicators related to nutrition and levels of pain/fear were also important to know for the assessment of welfare, with significant interactions between gender and stakeholder for both indicators (F_3_ = 3.76, *p* = 0.011 and F_3_ = 2.78, *p* = 0.040, respectively; Figure 2 and Figure 3). Stakeholder differences were noted for all welfare indicators (all *p* < 0.05) with the general public ranking indicators as being more important than other respondent groups (Table 4).

Respondents were given the opportunity to list any other indicators they perceived as important to know when assessing sheep welfare and a total of 348 (36%) respondents provided additional indicators. As these were open-ended responses the terms used and level of detail differed, with some respondents providing more than one indicator. The following comments were commonly perceived as being important welfare indicators: the provision of water (10.9%), improved transport conditions (10.1%), the cessation of/or better control over live export (9.5%), and the provision of shelter and/or shade (9.2%).

### 3.4. Self-Rated Knowledge

Participants were asked to rate their knowledge of the sheep industry. Overall average score for the self-rated understanding of the sheep meat industry was 3.63/5 (1 = poor understanding, 5 = very knowledgeable) and the wool industry was 3.58, with a very strong and highly significant correlation between self-rated understanding of both production systems (*r* (929) = 0.82, *p* < 0.001). Weak negative correlations were seen between a person’s belief about the importance of animal welfare and their understanding of the sheep meat industry (*r* (952) = −0.13, *p* < 0.001) and the wool industry (*r* (931) = −0.13, *p* < 0.001). Weak positive correlations were also seen between a person’s belief about the welfare of grazing sheep and their understanding of the sheep meat industry (*r* (926) = 0.24, *p* < 0.001) and the wool industry (*r* (905) = 0.28, *p* < 0.001). Mean knowledge scores and associated correlations for each stakeholder category are presented separately in Table 6. Producer self-rated understanding of each industry was weakly correlated with their beliefs about sheep welfare. An increased understanding of the sheep production industry was associated with the view that the welfare of sheep in extensive systems was poorer, but otherwise no correlations between the perception of sheep welfare, and the importance of animal welfare, were seen.

Respondents from the general public were also asked to rate their level of understanding on some specific sheep management issues (Table 7). The general public rated their understanding of both meat and wool industries, tail docking and crutching/shearing highest and nutritional requirements, parasite control and lambing lowest (F_9, 5086_ = 7.34, *p* < 0.001).

## 4. Discussion

The current study identified stakeholder perceptions of a variety of welfare issues facing extensively managed sheep. Stakeholder differences were present in three key responses: the perception of grazing sheep welfare, the importance of welfare issues (both specified and open-ended) and suitable key welfare indicators. The hypothesis was supported with the general public having a poorer perception of sheep welfare than producers, and the other two stakeholder groups having an intermediate perception. Additionally, there were gender differences throughout the results.

### 4.1. Stakeholder and Gender Influences

Across all groups, all welfare issues and indicators were considered to be important, with average scores across all categories being above the median. These results support the growing body of evidence that animal welfare is of broad societal concern [25]; however we acknowledge that there was a significant degree of bias in the surveyed population and we have addressed this with our statistical approach. There was a much higher representation of women in the general public category, which in a voluntary survey suggests a greater level of interest and care about the topic. It has been well demonstrated that women do indeed have a greater concern for animals and their welfare [28,29,30,31,32]. This was reflected in our survey with women rating welfare issues as being able to cause more compromise and they also considered all welfare indicators to be of greater importance when compared to men. Gender is an important consideration when evaluating other survey results; for example another survey conducted on welfare issues in extensively managed sheep also reported stakeholder differences [18], but gender was not further investigated as an underlying cause of stakeholder difference in that study.

A person’s attitude and beliefs are dependent on a variety of demographic, experiential and knowledge factors [5,33], and so it could be reasonably assumed that differences in knowledge of sheep may have driven the stakeholder differences. The general public had a low level of understanding about sheep production in the current study; a finding which is supported in the literature [14,17,34]. Although livestock welfare has been reported as an important issue for the general public in many different countries [35], it has been proposed that consumers with less knowledge of and experience with farming have a higher concern for welfare [20]. Without a reasonable level of understanding within which to place welfare issues and indicators [36] and a high degree of anthropomorphism [18], the general public may perceive all variables as being equally important. Given that knowledge is key in determining a person’s attitudes and beliefs it seems reasonable to assume that background knowledge, or the lack of background knowledge, may have affected perceptions. However, no conclusive relationships were seen between self-rated knowledge about the sheep meat or wool industries and the perceptions of sheep welfare and no relationships existed between perceived industry knowledge and the importance of animal welfare. The present evidence suggests that knowledge was not obviously associated with perception of sheep welfare. Essentially, people believe it to be important, regardless of how knowledgeable they are of the topic.

Other attitude modifiers consist of animal attributes, individual human attributes and cultural factors [5]. In this study we did not focus on animal attributes, nor did we identify individual human attributes such as early childhood experiences with animals, current interactions with animals, religiosity and personality [5]. The individual human attributes that were collected in this survey were gender, age, education, residence, knowledge, and of these factors, only gender was influential.

Understanding the role of our survey’s voluntary respondents in shaping societal views around animal welfare is an important step when considering the validity of these results for a broadly accepted welfare framework. Work by Coleman and colleagues [17] suggested that the most trusted sources of information about animal welfare, for the general public at least, included information received from friends and family, which suggests that the respondents to this survey may be an influential population. While our method for participant recruitment did not generate balanced demographics between stakeholder groups we believe that these results reflect those that are most interested in the topic, and so likely to be influencers around issues associated with sheep welfare and social licence. Future studies may use more targeted methods of recruitment to ensure an even distribution across genders. However, this may only be possible by setting quotas as voluntary surveys involving animal welfare issues tend to result in females being over-represented [17].

### 4.2. Welfare Issues

In the current study the importance of sheep welfare was endorsed by all stakeholders with all believing it to be of importance, and that there was the capacity for it to be compromised. There was a stakeholder × gender interaction in regard to the respondents’ perception of the welfare of grazing sheep with females from the general public stakeholder category believing welfare to be in more need of improvement than other groups. These findings are likely to be associated with the levels of understanding and knowledge of the general public, differences in perceptions between specific stakeholder groups such as the public and producers [9] as well as the higher levels of concern women have for the welfare of animals [28], a more negative view of animal use [30], animal husbandry systems and painful animal husbandry practices [29,37].

When assessing risks to sheep welfare, some commonalities were evident with respondents’ answers to the specified and open-ended questions. Of the specified issues, flystrike, nutrition and predation were considered most likely to compromise a sheep’s welfare, and all of these contribute to the PCA component named “environmental issues”. Participants also identified flystrike, other environmental factors, nutrition and predation to be major risks in the open-ended questions, although these may have been influenced by the specified issues posed in the earlier questions. The perceived significance of these environmental issues supports previous expert and producer studies which have also identified nutrition, predation and other environmental factors as issues that significantly influence the welfare of sheep. [3,6,16,38]. Compared to international papers [3,38], flystrike featured more prominently in the current results. Flystrike is a common regional issue for Australian sheep producers, and so the focus it received in this study is understandable.

There was also one very prominent response to the open-ended question, with issues around live export being identified as the single most significant issue facing sheep welfare. Examples of welfare compromise in live-exported sheep have been prominent in the last few years in Australia and have received a significant amount of media coverage. This likely influenced the views of the general public, who offered all responses on live export. While this was not identified as a key issue by the other stakeholder groups in the current survey, the live export of sheep has been previously identified as a significant welfare issue facing the sheep industry in a variety of reviews [39], industry expert and producer studies [6,16]. As this study was geared towards on-farm welfare, further consideration of this issue is not given here, but it highlights how issues within one area of the industry may affect broader perceptions, and thus may influence the social licence.

The results of the current survey show that the general public considered their understanding of both sheep meat and wool industries, tail docking and crutching/shearing highest, and nutritional requirements, parasite control and lambing the lowest. A survey by Phillips et al. [18] identified that stakeholders who were more removed from the production system ranked non-invasive issues, such as nutritional requirements and stockmanship, as lower welfare risks compared with more invasive practices like castration and tail docking. It is likely that these differences reflect the various perspectives and backgrounds of the stakeholder groups; animal health and productivity are likely to be of primary focus to a producer, while more injurious procedures are of immediate concern to the public as they are more visibly obvious. In this survey the self-rated knowledge of the public on general sheep husbandry did not influence what they considered to be the most important issues facing sheep (as determined by the open-ended welfare issues). An obvious limitation on further conclusions was that we assessed knowledge using a self-rated scale, which doesn’t necessarily reflect actual knowledge. Further assessment of actual knowledge compared to perceived knowledge would be important for future work in this area, particularly as education is one way that attitudes can be modified [33]. The Welfare Quality^®^ (WQ) framework consists of four principles of: good feeding, good housing, good health and appropriate behaviour [40]. These current results suggest that issues likely to compromise good feeding and housing (environmental issues and heat stress) were perceived to be more significant than those likely to compromise appropriate behaviour (husbandry practices).

### 4.3. Welfare Indicators

The ranking of welfare indicators showed that, in general, respondents found all listed indicators to be of some use when assessing the welfare of grazing sheep. All 17 of the indicators are found, in various forms, on the list of indicators suggested by a UK based expert group [3], used in the AWIN welfare assessment protocol for sheep [19], and mentioned by Goddard [41] in his review of assessing sheep welfare. This highlights a general agreement across a broader range of stakeholders than previously reported in the literature. Additionally, there is substantial alignment between the indicators that respondents perceive as important and the WQ framework. For example, the nutrition related PCA component included indicators such as change in liveweight and body condition score which aligns well with the WQ principle of good feeding. This provides further indication that the perceived key welfare indicators in a grazing environment found in this study are acceptable when compared to more well-developed assessment and monitoring protocols. However, it should be noted that many of these indicators have not been scientifically validated, with only limited data on repeatability and reliability for specific indicators such as body condition score [7].

The general public rated welfare indicators as more important than other stakeholders and an interaction was present between stakeholder and gender for the pain/fear and nutrition related composite scores. The pain/fear stakeholder × gender interaction showed that women from the general public believe this indicator to be more important when compared to other groups. As previously mentioned this corresponds with literature indicating that women place a greater significance on painful procedures in animals and rate occurrences of such procedures as being a significant compromise to welfare [29,37]. The stakeholder × gender interaction for the nutrition related composite score was somewhat different in that it appears to be due to the scientist group of stakeholders with male scientists rating nutrition to be of higher importance than the other male stakeholders while female scientists rated these indicators as lessor importance, particularly when compared with the female members of the general public. It is difficult to draw conclusions on this data and it is possible this inconsistency is an artefact caused by a biased sample population of scientists.

Welfare indicators are generally identified through a process involving a literature review, discussion with an expert group (e.g., industry based stakeholders such as producers, veterinarians and industry service providers) and scientific testing [3,19]. This survey has taken a different approach and listed what appear to be the most valuable welfare indicators taken from a variety of sources (literature, industry consultants, and others involved in the sheep industry) and then attempted to confirm whether or not a broader group of stakeholders believe them to be of value. It is unlikely that consumers will be interested in the specific detail involved in a sheep production system [20]; however involving all stakeholders along the food supply chain and, if possible, gaining a consensus of useful measurement parameters may increase the acceptance of a food production system and trust between stakeholders.

## 5. Conclusions

Sheep welfare was an important consideration to all stakeholders in this survey, and all issues were perceived to cause some degree of welfare compromise. Welfare issues of key importance to the stakeholders were those relating to environmental issues, heat stress, lameness and husbandry practices. The welfare indicators perceived to be of key importance were those related to nutrition, pain and fear, mortality and management, feed on offer and the number of illness/injuries occurring within a flock. Both gender and stakeholder differences were clear, the most notable were women’s greater concern for welfare and the general public’s concern for off-farm issues. These results highlight the importance of including all stakeholders and an even balance of genders when developing a welfare framework that can address both practical and societal concerns.

## Figures and Tables

**Table 1:** Specific questions and response options for the topics “demographics”, “knowledge and beliefs” and “risks to sheep welfare” and “key indicators” investigated in the survey.

Question Category	Stakeholder Group	Specific Question	Answer Options
Demographics	All	Gender	
		Highest level of education	No formal schooling; Primary school; High school; Technical or further educational institution (incl. TAFE); University or higher
		Year of birth	
		State of Australia	
		Current residential location	Urban; Suburban; Peri-urban; Regional city; Country town; Rural; Remote
Knowledge and beliefs	All	What is your belief about animal welfare?	An insignificant issue; Of minor importance; Of moderate importance; Of major importance
		I think the welfare of grazing sheep in Australia is…	Very poor; Poor with many areas for improvement; Generally adequate but some areas could be improved; Adequate; Excellent
		How would you rate your understanding of Australian sheep production systems?: The sheep meat industry The wool industry	Poor; Limited; Moderate; Knowledgeable; Very knowledgeable
	General public	How would you rate your understanding of the following sheep-related management practices in Australia?: Parasite control Lambing Mulesing Tail docking Castration Crutching/shearing Nutritional requirements General sheep husbandry	Poor; Limited; Moderate; Knowledgeable; Very knowledgeable
Factors affecting sheep welfare	All	Rate how important the following issues can be in compromising the welfare of grazing sheep in Australia: Cold stress Disease Flystrike Heat stress Painful husbandry procedures (excluding mulesing) Internal parasite burden Lambing difficulties Lameness Mental state of the animal Mulesing Nutrition and food supply Poisonous plants Predation by dogs, pigs, foxes Pregnant ewe body condition Pregnant ewe health Road transport Shearing/crutching Use of sheep dogs Weekly monitoring Yarding	Number from 1–5, with 1 = no compromise through to 5 = extreme compromise
		In your opinion, what are the three most important welfare issues in the Australian sheep industry? List in order of importance. You can use those from the question above or add your own.	Open-ended responses
Welfare indicators	All	What would you want to know if you were to assess the welfare of sheep?: Changes in body condition score Changes in liveweight Environmental conditions Ewe mortality across the whole flock Fearfulness (distance of flight zone) Feed on offer Frequency of monitoring Internal parasite burden Lamb mortality across the whole flock Level of pain mitigation used during husbandry procedures associated with pain Mental state of the animal Number of illness/injuries in a flock Occurrences of husbandry procedures associated with pain Occurrences of lameness Severity of illness/injuries to individual animals Stocking density under grazing conditions Stockmanship skill level	Number from 1–5, with 1 = unimportant through to 5 = essential

**Table 2:** Principal components analysis and variables for the survey questions.

Question	Component	Variables	Variance Accounted (%)	Eigenvalue (%)
Welfare issues	Environmental issues	Cold stress	58.7	11.7
		Disease		
		Flystrike		
		Internal parasite burden		
		Lambing difficulties		
		Nutrition and food supply		
		Poisonous plants		
		Predation by dogs, pigs, foxes		
		Pregnant ewe body condition		
		Pregnant ewe health		
	Husbandry practices	Mental state of the animal	9.2	1.8
		Mulesing		
		Painful husbandry procedures (excluding mulesing)		
		Road transport		
		Shearing/crutching		
		Use of sheep dogs		
		Weekly monitoring		
		Yarding		
	Independent	Lameness		
	Independent	Heat stress		
Welfare indicators	Pain and fear	Fearfulness (distance of flight zone)	50.8	8.6
		Frequency of monitoring		
		Level of pain mitigation used during painful procedures		
		Mental state of the animal		
		Occurrences of procedures associated with pain		
		Occurrence of lameness		
		Severity of illness/injuries to individuals		
	Nutrition	Changes in body condition score	9.8	1.7
		Changes in live weight		
		Environmental conditions		
	Mortality and management	Ewe mortality rate across flock	4.8	0.8
		Internal parasite burden		
		Lamb mortality rate across flock		
		Stocking density under grazing conditions		
		Stockmanship skill level		
	Independent	Feed on offer		
	Independent	Number of illness/injuries in the flock		

**Table 3:** Gender-based differences in beliefs about animal welfare, specific welfare issues and indicators used to assess welfare. Means with standard deviations presented in parentheses.

Topic		Gender		Overall
		Female	Male	
Belief about animal welfare ^1^		3.88 (±0.43)	3.61 (±0.69)	3.78 (±0.07)
Welfare issue ^2^	Environmental	3.98 (±0.98)	3.69 (±0.78)	3.87 (±0.12)
	Husbandry practices	3.71 (±1.11)	2.85 (±0.89)	3.37 (±0.13)
	Heat stress	4.14 (±1.17)	3.23 (±1.08)	3.79 (±0.15)
	Lameness	3.81 (±1.15)	3.22 (±1.05)	3.57 (±0.15)
Welfare indicator ^3^	Management issues	4.44 (±0.65)	4.01 (±0.70)	4.27 (±0.09)
	Food on offer	4.55 (±0.65)	4.19 (±0.70)	4.41 (±0.10)
	Number of illness/injuries in the flock	4.58 (±0.73)	3.93 (±0.96)	4.33 (±0.12)

**^1^** Responses could range from 1 = insignificant to 4 = major importance; **^2^** Responses could range from 1 = no compromise to welfare to 5 = significantly compromises welfare; **^3^** Responses could range from 1 = unimportant to know to 5 = essential to know.

**Table 4:** Stakeholder-based differences in beliefs about animal welfare, specific welfare issues and indicators used to assess welfare. Means with standard deviations presented in parentheses.

Topic		Stakeholder Group				Overall
		Producers	Service Providers	Scientist	General Public	
Belief about animal welfare ^1^		3.65 ^b,^* (±0.70)	3.63 ^b^ (±0.53)	3.69 ^b^ (±0.49)	3.88 ^a^ (±0.46)	3.78 (±0.17)
Welfare issue ^2^	Environmental	3.62 ^b^ (±0.77)	3.88 ^ab^ (±0.68)	3.91 ^a^ (±0.71)	3.98 ^a^ (±1.02)	3.87 (±0.27)
	Husbandry practices	2.73 ^c^ (±0.90)	2.31 ^b^ (±0.80)	3.09 ^b^ (±0.86)	3.83 ^a^ (±1.10)	3.37 (±0.30)
	Heat stress	3.14 ^c^ (±1.07)	3.38 ^bc^ (±1.01)	3.57 ^b^ (±1.05)	4.24 ^a^ (±0.78)	3.79 (±0.34)
	Lameness	3.05 ^c^ (±1.04)	3.46 ^b^ (±0.90)	3.49 ^b^ (±1.04)	3.90 ^a^ (±1.16)	3.57 (±0.33)
Welfare indicator ^3^	Management issues	4.06 ^b^ (±0.72)	4.02 ^b^ (±0.65)	3.95 ^b^ (±0.69)	4.49 ^a^ (±0.63)	4.27 (±0.20)
	Food on offer	4.31 ^b^ (±0.77)	4.37 ^ab^ (±0.87)	4.12 ^b^ (±0.93)	4.52 ^a^ (±0.76)	4.41 (±0.24)
	Number of illness/injuries in the flock	3.97 ^b^ (±0.96)	3.99 ^b^ (±0.99)	4.10 ^b^ (±0.96)	4.65 ^a^ (±0.68)	4.33 (±0.25)

**^1^** Responses could range from 1 = insignificant to 4 = major importance; **^2^** Responses could range from 1 = no compromise to welfare to 5 = significantly compromises welfare; **^3^** Responses could range from 1 = unimportant to know to 5 = essential to know; * Values with different superscripts (**^a^**, **^b^**, **^c^**) indicate statistically significant differences within rows at *p* < 0.05.

**Table 5:** The most important sheep welfare issue according to each stakeholder and gender; responses are provided as a % for each gender in each stakeholder group.

Issue	Stakeholder	Gender	Disease/Illness	Environmental	Injury/PHP	Management	Nutritional	Off Farm	Other
Issue 1	Producer	Female	26.4	6.9	25	13.9	4.2	9.7	8.3
		Male	34.7	9.5	24.2	7.9	8.9	4.2	7.4
	Service provider	Female	22.7	0	45.5	4.5	9.1	9.1	9.1
		Male	30.8	6.2	29.2	7.7	9.2	9.2	3.1
	Scientist	Female	48.8	7	16.3	2.3	4.7	18.6	2.3
		Male	34.6	1.9	28.8	9.6	13.5	3.8	5.8
	General public	Female	7.7	4.6	20.1	4.4	5.3	48.7	7.1
		Male	10	5	23.3	11.7	5	33.3	10
Issue 2	Producer	Female	18.1	8.3	22.2	13.9	4.2	20.8	9.7
		Male	28.4	5.3	24.7	8.9	7.4	12.6	7.4
	Service provider	Female	27.3	9.1	27.3	18.2	4.5	13.6	0
		Male	32.3	7.7	16.9	6.2	18.5	12.3	4.6
	Scientist	Female	16.3	7	46.5	14	4.7	2.3	4.7
		Male	28.8	7.7	28.8	9.6	5.8	9.6	5.8
	General public	Female	6.9	10.8	26.3	9.5	4	28.3	9.5
		Male	13.3	20	21.7	6.7	3.3	20	8.3
Issue 3	Producer	Female	6.9	9.7	26.4	20.8	2.8	11.1	12.5
		Male	24.7	7.4	18.9	16.3	4.7	5.3	12.1
	Service provider	Female	13.6	0	31.8	4.5	27.3	4.5	13.6
		Male	30.8	13.8	24.6	1.5	13.8	3.1	7.7
	Scientist	Female	25.6	4.7	23.3	9.3	14	9.3	9.3
		Male	21.2	7.7	32.7	7.7	15.4	7.7	5.8
	General public	Female	8	9.5	25.2	12.8	7.7	21	10.6
		Male	6.7	11.7	21.7	8.3	5	21.7	15

**Table 6:** The mean scores and Pearson’s correlations for the self-rated understanding on sheep production and beliefs and the welfare of grazing sheep; 1 = poor understanding, 5 = very knowledgeable.

Stakeholder	Self-Rated Knowledge	Mean Score	Pearson’s Correlations *		
			Understanding of the Wool Industry	Belief about Animal Welfare	Belief about the Welfare of Grazing Sheep
Producer	Sheep meat industry	4.07	0.50 (<0.001)	−0.076 (0.22)	0.13 (0.034)
	Wool industry	3.98		−0.058 (0.363)	0.23 (<0.001)
Industry service provider	Sheep meat industry	4.46	0.54 (<0.001)	0.023 (0.83)	0.19 (0.08)
	Wool industry	4.44		0.153 (0.156)	0.035 (0.746)
Sheep specific scientist	Sheep meat industry	4.2	0.670 (<0.001)	0.075 (0.47)	0.026 (0.80)
	Wool industry	4.19		−0.11 (0.30)	0.10 (0.33)
General public	Sheep meat industry	3.15	0.89 (<0.001)	−0.046 (0.30)	0.009 (0.85)
	Wool industry	3.11		−0.06 (0.18)	0.068 (0.14)

* *r* values are reported with *p* values presented in the parentheses.

**Table 7:** The mean scores of the General Public’s self-rated understanding of sheep management; 1 = poor understanding, 5 = very knowledgeable.

Management Issue	Mean Score/5 *
The sheep meat industry	3.15 ^a^
Tail docking	3.12 ^a^
The wool industry	3.11 ^a,b^
Crutching/shearing	3.11 ^a^
Castration	3.07 ^a,d^
Mulesing	2.97 ^b,c,d,e^
General sheep husbandry	2.87 ^c,e^
Lambing	2.86 ^c^
Parasite control	2.84 ^c^
Nutritional requirements	2.81 ^c^
Pooled standard error of the mean	0.16

* Values with different superscripts (**^a–e^**) indicate significant differences between rows at *p* < 0.05.
